# Are mental health and binge drinking associated in Dutch adolescents? Cross-sectional public health study

**DOI:** 10.1186/1756-0500-4-100

**Published:** 2011-04-04

**Authors:** Marie-José Theunissen, Maria Jansen, Anke van Gestel

**Affiliations:** 1 GGD Brabant Zuidoost, P.O Box 810, 5700 AV Helmond, the Netherlands; 2 Maastricht University, CAPHRI, Department of Health Promotion P.O. Box 616 6200 MD Maastricht, the Netherlands

## Abstract

**Background:**

Depression and anxiety disorders have a high disease burden and as many as 15% of young people report mental health problems. Binge drinking, which is a particularly harmful way of consuming alcohol, is common among secondary school students. The aim of this study was to examine the association between binge drinking and self-reported mental health in boys and girls aged 12 to 18 years.

**Findings:**

This cross-sectional analysis was performed on data collected by the Community Health Service (GGD) Brabant Zuidoost, the Netherlands, in 2007. In this Youth Survey, 10 090 randomly selected adolescents aged 12 tot 18 years were each sent a letter, a questionnaire, and a user name and log-in code for if they preferred to complete the Internet version of the questionnaire. Mental health was assessed using the Mental Health Inventory (MHI-5), a short 5-item questionnaire to detect feelings of depression and anxiety. Participants were asked about current alcohol consumption, their relationship with their parents, drug use, and sociodemographic data.

Corrected for confounders, binge drinking and mental health problems were associated in the 12 to 15 year old girls (OR 2.43; 95% CI 1.86-3.17, p = 0.000) and boys (OR 1.64, 95% CI 1.19-2.27, p = 0.003). The majority of the 16 to 18 year old adolescents had been binge drinking in the previous 4 weeks (69.6% boys and 56.8% girls). In this age group, boys with mental health problems were less likely to be classified as binge drinkers than were boys without mental health problems (OR 0.63, 95% CI 0.45-0.87, p = 0.005). No such association between binge drinking and mental health was found in girls of this age.

**Conclusion:**

Girls and boys aged 12-15 years were classified as binge drinkers significantly more often when they reported poor mental health. Because binge drinking damages the brain, especially at a young age, it is important that health professionals are alert to possible binge drinking when young adolescents report mental health problems and should ask their patients about their drinking behaviour. Likewise, if youngsters under 16 present with binge drinking, they should be asked whether they are anxious or depressed.

## Background

Adolescence is not only a transitional stage between childhood and adulthood, it is also a critical phase in brain development and maturity during which the brain undergoes progressive and regressive changes [1,2]. Excessive alcohol use at a young age can disturb this process and can seriously damage the development of the brain [3,4]. Binge drinking in particularly is a harmful way to consume alcohol [5,6]. During puberty, adolescents exhibit risk behaviour [7-9], such as drinking too much, [10-13], and studies have shown that excessive alcohol consumption in adolescents is strongly related to problem behaviour and an increased risk of suicidal behaviour [14,15], with six or more glasses of alcohol weekly being associated with an increased risk of depression [16]. Moreover, boys with clinical depression start drinking alcohol at a younger age than their non-depressed peers [17,18]. Subclinical mood changes are common in adolescence, ranging from "dips" in mood that usually last no longer than a few weeks to subclinical depression, which affects 17% of young people [19]. Depression and anxiety disorders have a high disease burden, even in a mild form [20] and teenagers with subclinical depression have a 6 times higher risk of developing clinical depression than teenagers without subclinical depression [19], and adolescents diagnosed with a depressive disorder are at higher risk of substance abuse, future depression, and suicidal behaviour [14,21,22]. Fifteen percent of adolescents report anxiety [23], with social phobia and generalized anxiety disorder in particular developing during childhood and adolescence [24]. These young people are at increased risk of developing other anxiety disorders, depression, and substance dependence [25]. Indeed, current anxiety is strongly associated with alcohol abuse in adolescents seen in primary care settings [26].

Although the number of binge drinking youngsters aged 12-14 years has decreased in recent years from 28% in 2003 to 19% in 2007, the proportion of binge drinking students aged 15-16 years has remained stable (57%). Compared with their peers in other European countries, Dutch students can be considered heavy drinkers [27-29]. In the Netherlands, the Youth Health Service provides the parents and guardians of children aged 4-18 years with guidance regarding the physical, mental, and social development of their children. One of the Service's primary tasks is to identify health risks at an early stage, which necessitates monitoring mental health and lifestyle risks, including alcohol consumption. Little is known about the drinking behaviour, and especially binge drinking, of secondary school students who have moderate or poor mental health but who have not been clinically diagnosed with depression or anxiety disorder. The aim of this study was to establish whether self-reported moderate or poor mental health is associated with binge drinking in boys and girls aged 12 to18 years.

## Methods

### Participants and procedures

The study was carried out by the Community Health Service (GGD) Brabant-Zuidoost, the Netherlands. Data for this study were obtained from "jeugdmonitor 12 t/m 18 jarigen 2007, GGD Brabant Zuidoost", the Provincial Youth Survey held in the south-east of the Netherlands in November 2007. This was a cross-sectional survey with self-administered questionnaires held among 19 386 youngsters aged 12 to 18 years on 1 October 2007 and living in the province of North Brabant, in the south-east part of the Netherlands. The adolescents were randomly selected, stratified by municipality, using the software application of "Statistical Package for Social Sciences (SPSS 16). The name and date of birth of the adolescents were obtained from the personal records database of the municipality.

All adolescents were sent, to their home address, a letter containing a user name and log-in code for filling in the Internet-based questionnaire, and a paper version of the questionnaire, for those without access to Internet, plus a stamp-addressed envelope for returning the completed questionnaire. The letter was addressed to the parents, who implicitly agreed to their child's participation if the child completed the questionnaire. After 6 and 12 weeks, non-responders received a reminder and a new user name, log-in code, and the paper version of the self-administered questionnaire. A raffle was held among participants, with prizes worth €15. Ethical review was not necessary for the secondary analysis of anonymous data.

### Measures

#### Mental health

Mental health was measured with the Mental Health Inventory (MHI-5), a brief 5-item questionnaire [30]. The MHI-5 measures general mental health and can be used to screen for depressive symptoms and feelings of anxiety [31,32]. It is part of the Short Form Health Survey (SF-36) [33,34]. The MHI-5 compromises five questions (Table 1), each with six possible response, scored between 1 and 6 (total score ranges from 5 to 30). The total score is transformed into a variable ranging from 0-100 using a standard linear transformation, with a score of 100 representing optimal mental health. Because no formal cut-off point has been determined for the Dutch version of the MHI-5, we used a cut-off score of 60 to define moderate-to-poor mental health. This cut-off is widely used and provides the best sensitivity and specificity for detecting depressive symptoms [35].

**Table 1:** Questions and response categories of the MHI-5.

MH-5 score is based on the following five questions:*		
How much of the time in the previous 4 weeks:		
a. Have you been a very nervous person?		
b. Have you felt so down in the dumps that nothing could cheer you up?		
c. Have you felt calm and peaceful?		
d. Have you felt downhearted and blue?		
e. Have you been a happy person?		
		
**Response category**	**Score on questions a, b, d**	**Score on questions c, e**

1. All of the time	1	6
2. Most of the time	2	5
3. A good bit of time	3	4
4. Some of the time	4	3
5. A little of the time	5	2
6. None of the time	6	1

* The score from the MHI-5 is computed by adding the scores for each question item and then transforming the raw scores to a 0-100 point scale. Total score on MHI-5 = 100*((score a +score b + score c + score d + score e)-5)/25

#### Binge drinking

In this study, binge drinking was defined as 5 or more alcoholic drinks consumed on one occasion, a definition commonly used in Europe [23,36]. The National Institute on Alcohol Abuse and Alcoholism (NIAAA) defines binge drinking as an increase in blood alcohol level of 0.8 or higher [37]. For adults, this means drinking 5 or more "standard" drinks within 2 hours for men, and 4 or more within 2 hours for women. However, there is no international consensus on the number of drinks or on the time within which the alcohol should be consumed. Moreover, the amount of alcohol in a standard drink varies by country.

Participants in this study were asked to report the number of times they consumed 5 or more drinks on one occasion (one evening or at a party) in the past 4 weeks. To derive the frequency of alcohol consumption and the average quantity of alcohol consumed, participants were asked to indicate the usual number of weekdays and weekend days per week they drunk alcohol and the number of drinks usually consumed per drinking day. Participants also reported whether they sometimes used drugs and alcohol together. For this study, the answers were recoded into two categories (binge drinker yes/no).

#### Confounding factors

The following factors were considered to be potential confounders: age (recoded into the category '12 to 15' and '16 to 18 years'), sex, ethnicity, family situation (living or not living with both parents), the participant's relationship with their parents (below average), education level (recoded into lower general secondary education, and higher general secondary education), stressful life events, having ADHD, and frequency of drug use (cannabis).

#### Data analysis

A weighting procedure was used to enable generalization of the findings to the general adolescent population in the south-east Netherlands. Post-stratification weights were calculated by comparing sample distributions and known population distributions of sex, age, and town size (the national statistics were obtained from the Central Bureau for Statistics; see http://www.cbs.nl). Non-response to a particular question was treated as missing for all analyses using that variable. Descriptive statistics for the total sample, including frequencies, means, and standard deviations (SD), were calculated. Analyses were stratified by age (12-15 and 16-18 years) and sex. Chi-square tests were performed (2 × 2 contingency tables) with binge drinking and all potential confounders and with mental health and all potential confounders (e.g., binge drinking or mental health and drugs, binge drinking or mental health and ADHD, binge drinking or mental health and ethnicity etc.), to determine which factors were confounders. Differences were tested for significance (p < 0.05). Odds Ratios (OR) and Likelihood ratios were calculated.

To investigate the association between binge drinking and mental health, multivariate logistic regression analyses were conducted with binge drinking as dependent variable and mental health as independent variable, controlling for confounders. The Forced Entry method was used, so that all predictor variables were tested in one block to assess their predictive ability, while controlling the effects of other predictors in the model. Within the logistic regression interactions and significance are examined. As regression analysis indicated that age and sex were significant predictors of both binge drinking and mental health, interaction terms were calculated for these variables.

A-2 log likelihood test with maximally 20 iterations was performed. A variable was entered into the model if the probability of its score statistic was less than the Entry value (= 0.05) and was removed if the probability was greater than the removal value (= 0.10). Cases with predicted values that exceed the classification cutoff (= 0.5) are classified as positive, while those with predicted values smaller than the cutoff are classified as negative. All statistical analyses were performed using SPSS 16.0

## Results

The response rate was 52%; 77% of the participants completed the paper version of the questionnaire and 23% the Internet version.

Data collected about the frequency of mental health problems and binge drinking, socioeconomic variables, and possible confounders are given in Table 2.

**Table 2:** Sample characteristics.*

Age in years		12-15		16-18	
	Total sample	Male	Female	Male	Female
N not weighted	10 019 (100)	2880 (46.9)	3260 (53.1)	1680 (43.3)	2199 (56.7)
					
N weighted**	10 020 (100)	2927 (51.0)	2808 (49.0)	2192 (51.2)	2093 (48.8)
Ethnic groups, non-western***	368 (3.7)	113 (3.9)	116 (4.2)	69 (3.1)	70 (3.4)
School, VMBO****	2274 (22.7)	974 (42.6)	924 (40.9)	215 (17.3)	161 (14.0)
Family, not living with both parents	1580 (15.8)	428 (15.3)	466 (17.2)	352 (17.1)	334 (17.5)
Parents, relationship below average	1866 (18.6)	434 (14.9)	484 (17.3)	454 (20.8)	494 (23.6)
Problematic life-events	2093 (20.9)	455 (15.6)	669 (23.9)	403 (18.5)	566 (27.2)
ADHD	219 (2.2)	106 (3.8)	18 (0.7)	63 (3.0)	32 (1.6)
Binge drinking previous 4 wks	3393 (33.9)	372 (12.9)	339 (12.3)	1506 (68.7)	1176 (56.8)
Binge drinking previous 4 wks > = 3 times	1607 (16.0)	97 (3.3)	90 (3.2)	904 (41.8)	516 (24.9)
Soft drugs previous 4 wks	398 (4.0)	45 (1.6)	34 (1.3)	203 (9.4)	116 (5.6)
Alcohol and drugs together	342 (3.4)	37 (1.3)	25 (0.9)	183 (8.4)	97 (4.7)
Mental health, moderate or poor	1389 (13.9)	247 (8.6)	418 (15.0)	259 (11.9)	465 (22.5)

* Data are shown as number and valid percentage (%) for the total sample and for sex within age strata.

** A weighting procedure was applied for gender, age and town size.

*** Non-western ethnic groups include immigrants from Turkey, Morocco, Surinam, Aruba and the Netherlands Antilles, Africa, Asia (Indonesia and Japan not included) and Latin America. Western ethnic groups include immigrants from: European countries (Turkey not included) Indonesia, Japan, North America and Oceania.

**** VMBO, preparatory secondary vocational education, it is a preparation for middle-level applied education.

Girls reported poor or moderate mental health more often than did boys. The older boys and girls were, the more often they reported mental health problems, with the reporting rate increasing from 9.0% in 12-year-olds to 18.4% in 18-year-olds. Most of the adolescents aged 16-18 years had been binge drinking in the previous 4 weeks. The proportion of adolescents with mental health problems who were binge drinkers is given in Table 3.

**Table 3:** Chi-square tests: Association between mental health problems and binge drinking.*

Age in years		12-15		16-18	
	Total sample	Male	Female	Male	Female
Mental health problems and binge drinking	538(39.2)	44(18.6)	95(23.0)	150(59.5)	244(53.0)
No mental health problems and binge drinking	2847(33.6)	327(12.6)	240(10.3)	1344(71.0)	914(57.6)
Odds Ratio** (CI)	1.3(1.1-1.4)	1.6(1.1-2.2)	2.6(2.0-3.4)	0.6(0.5-0.8)	0.8(0.7-1.0)
Likelihood Ratio***	16.4	6.2	45.4	13.2	3.0
	p = 0.000	p = 0.013	P = 0.000	P = 0.000	p = 0.081(ns)

* Data are shown as weighted frequencies and valid percentage (%) for the total sample and for sex within age strata.

**Crude OR

*** df are all 1

Girls and boys aged 12-15 years with mental health problems were more likely to be binge drinkers than were their peers without mental health problems. However, boys aged 16-18 years with mental health problems were less likely to be binge drinkers than their peers without mental health problems. Their was no such significant relationship in 16-to 18- year old girls.

Table 4 shows the results of the logistic regression analysis with binge drinking as dependent variable. Girls aged 12-15 were 2.4-times more likely to be binge drinkers if they reported moderate or poor mental health, even after correction for education, ethnicity and family situation. Boys of the same age were 1.6 times more likely to be binge drinkers if they reported mental health problems after correction for potential confounders. In contrast, boys aged 16-18 years reported binge drinking significantly less often in the past 4 weeks if they experienced moderate or poor mental health, they were 0.6 times less likely to be classified as binge drinkers if they experienced mental health problems. No such association was seen in girls of the same age.

**Table 4:** Logistic regression analysis of the relationship between binge drinking (dependent variable) and mental health problems,

Age (years)		B	**S.E**.	Wald*	**Sign**.	Exp(B)**	95% CI for Exp(B)
12-15	Male	0.50	0.17	8.92	0.003	1.64	1.19-2.27
	Female	0.89	0.14	42.68	0.000	2.43	1.86-3.17
							
16-18	Male	-0.47	0.17	7.96	0.005	0.63	0.45-0.87
	Female	-0.08	0.14	0.29	0.590 (ns)	0.93	0.70-1.22
							

**-2 Log Likelihood**			**Cox & Snell R Square**			**Nagelkerke R Square**	

6313.901***			0.232			0.329	

* df are all 1

** Adjusted for age, sex, education (VMBO; preparatory secondary vocational education), ethnicity (western ethnic groups), family situation (not living with both parents)

***Estimation terminated at iteration number 5 because parameter estimates changed by less than 0.001.

## Discussion

The purpose of this study was to investigate whether self-reported moderate or poor mental health is associated with binge drinking in boys and girls aged 12-18 years. We found that boys and girls aged 12-15 years with mental health problems were at significantly higher risk of binge drinking than their mentally well peers and this association remained after correction for confounders. The majority of boys aged 16-18 years were binge drinkers (69.6%). In the Netherlands, the legal age for buying drinks containing less than 15% alcohol is 16 years (1964 Licensing and Catering Act). Most youngsters go to the pub once they become 16, and most drink at home with friends before going out [12]. Drinking is a social activity. While it has been reported that binge drinking boys exhibit externalizing behaviour [15], we found that the boys aged 16-18 years with mental health problems were significantly less likely to be binge drinkers. A possible explanation is that these boys tend to withdraw when experiencing social phobia or depressive feelings, so they are more likely to stay at home than to go out drinking with friends. We did not find mental health problems to be associated with binge drinking in girls aged 16-18 years. This is contrary to our expectation, because alcohol consumption is reported to increase in female students who experience daily sadness [38]. This discrepancy might be due to differences in study population or as a result of selection bias (relatively many mentally well girls might have completed the questionnaire). Further research is recommended.

### Strengths and limitations

The large sample size (10 090 adolescents) of this study made it possible to stratify by age and sex. We chose to stratify by age (under 16 and 16 years and older) because 16 is the legal age for buying alcohol in the Netherlands and because binge drinking and mental health problems are strongly associated with age. We investigated the frequency of binge drinking in adolescents aged 12-18 years with or without mental health problems for each age group separately, but because few children aged 12-13 years were binge drinkers and had mental health problems, we could not make comparisons for each age group. However, we did find binge drinking to be associated with self-reported mental health problems in children younger than 16 years. In contrast, binge drinking was not associated with mental health problems in children older than 16 years, in the sense that in this age group it was typically children without mental health problems who were binge drinkers.

The administration of the questionnaires at home and the assurance that data would be processed anonymously might have encouraged the participants to respond truthfully, so that we collected reliable and valid data. It can be expected that peer influence is less when questionnaires are completed at home rather than at school. A potential limitation of this study is the reliance on self-report data, so that responses to sensitive questions about undesirable or illegal behaviour may have been biased. Another potential limitation is the seemingly low response rate of 52%. We did not perform a non-responder analysis, and so we do not know whether heavy drinkers or adolescents with poor mental health were under-represented or over-represented in our sample. We think that binge drinking among adolescents with mental health problems is more likely to have been underestimated than overestimated in our study, because both mental health problems and binge drinking increase with age and our study included more younger than older adolescents. Moreover, Knudsen et al. showed that the non-participants in their population-based health study typically had poorer health habits (including risky alcohol consumption) and poorer general somatic and mental health than the participants. These authors also showed that internal associations tended not to vary much with response rate [39]. In a review article published in 2007, Galea and Tracy also concluded that the available empirical findings showed "little evidence for substantial bias as a result of nonparticipation" [40].

There may also have been a selection bias because few immigrants and more adolescents who were following higher general secondary education than lower general secondary education participated in the study. Moreover, more girls than boys and more young adolescents than older adolescents participated; however, we attempted to correct for this by using a weighting procedure for sex and age. Lastly, the cross-sectional design limited the ability to draw conclusions about causality.

## Conclusions

Anxiety and depression are major causes of morbidity and disability and constitute a major public health burden [20]. Binge drinking is a particularly harmful way to consume alcohol [6], and especially at a young age when the brain is still developing and is susceptible to alcohol-induced damage [4,5]. Different definitions of binge drinking are used in the literature, but we defined binge drinking as the consumption of 5 or more alcoholic drinks on one occasion. As the blood alcohol level increases when alcohol is drunk over a short period, other definitions of binge drinking that specify the period over which the drinks are consumed (such as the NIAAA) may result in a stronger or weaker association between binge drinking and poor mental health.

The association between mental health and binge drinking emphasizes the importance of alertness on the part of primary care practitioners and youth health services to depressive and anxiety symptoms and binge drinking, especially among boys and girls aged 12-15 years. In the Netherlands, all youngsters in the second class of secondary school, when most children are 13-15 years old, are given a medical examination by the youth health services. If adolescents, especially girls, report moderate or poor mental health, they should be asked about their drinking habits and, vice versa, if they report binge drinking, they should be asked whether they are anxious or depressed.

Many professionals are not aware of the association between mental health problems and alcohol use [41]. Yet knowledge of the risk of comorbidity between anxiety or depressive feelings and binge drinking in adolescents, and early identification of those at risk, will lead to early and more effective intervention. Effective interventions for youths are described in the database of the Netherlands Youth Institute (NJI); see http://www.nji.nl.

A longitudinal study of the association between mental health and binge drinking is needed to understand the causal link between binge drinking and mental health problems, which may help to improve the early detection and treatment of both problems.

## Competing interests

The authors declare that they have no competing interests.

## Authors' contributions

MT designed the study, wrote the protocol, conducted literature searches, provided summaries of previous research studies, performed the statistical analysis and drafted the manuscript. MJ participated in the design of the study and the protocol. AvG conducted the statistical analysis. All authors read and approved the final manuscript.
